# Intracranial Solitary Fibrous Tumor/Hemangiopericytoma: A Series of 14 Cases and Review of the Literature

**DOI:** 10.7759/cureus.59798

**Published:** 2024-05-07

**Authors:** Corneliu Toader, Dorel Arsene, Andrei Adrian Popa, Razvan-Adrian Covache-Busuioc, Bogdan-Gabriel Bratu, Luca-Andrei Glavan, David-Ioan Dumitrascu, Alexandru Vladimir Ciurea

**Affiliations:** 1 Department of Neurosurgery, “Carol Davila” University of Medicine and Pharmacy, Bucharest, ROU; 2 Department of Anatomical Pathology, “Carol Davila” University of Medicine and Pharmacy, Bucharest, ROU

**Keywords:** histological results, neurological outcomes, surgical treatment, hemangiopericytoma, intracranial solitary fibrous tumor

## Abstract

Solitary fibrous tumor (SFT) is a rare type of tumor characterized by spindle-shaped cells originating from mesenchymal tissue. This case series presents a collection of 14 intracranial solitary fibrous tumors treated between 2014 and 2022 in our institute in Bucharest, Romania. Through a systematic investigation, key aspects spanning the preoperative, intraoperative, and postoperative phases of patient care were highlighted. Our study examines various factors including tumor location (which was very heterogeneous), size (median of 49 mm, ranging between 22 mm and 70 mm), surgical techniques employed, and recurrence rates. The data was analyzed using Python version 3.10 (Python Software Foundation, Wilmington, Delaware, United States). Gender disparities in SFT were noted, particularly the male-to-female ratio which was 5:9. The use of the Medical Research Council (MRC) Scale for Muscle Strength aided in evaluating severity and postoperative outcomes. GTR was achieved in nine out of 14 cases (64.28%), prolonging the period of recurrence-free survival.

## Introduction

Solitary fibrous tumor (SFT) represents an uncommon neoplasm characterized by spindle-shaped cells and derived from mesenchymal tissue. Predominantly manifesting in the pleura and peritoneum, SFT can also present in various extra-pleural organs and tissues [1]. Since its initial identification within the central nervous system (CNS) by Carneiro et al. in 1996, the occurrence of intracranial solitary fibrous tumor (ISFT) has been increasingly reported. Despite this, the imaging characteristics of ISFT frequently exhibit a lack of specificity [2].

The 2007 edition of the WHO Tumor Classification for the CNS differentiated SFT and hemangiopericytomas (HPC) as distinct entities [3]. However, advancements in genetic profiling and immunohistochemical analysis have revealed that SFTs and HPCs share morphological similarities and are characterized by the presence of the 12q13 chromosome region and the fusion of the NAB2 gene with the signal transducer and activator of transcription 6 (*STAT6*) gene [4]. This fusion leads to the pathological and immunohistochemical reversal, manifesting as nuclear STAT6 expression. Consequently, the distinction between the diagnoses of these two tumor types has become increasingly blurred [5].

In 1942, Stout and Murray coined the term "hemangiopericytoma" for a tumor originating from pericytes, the contractile cells surrounding capillaries and postcapillary venules [6]. This neoplasm, which is both mesenchymal and highly vascularized, is located in the arachnoid layer of the intracranial space, a characteristic it shares with meningiomas. However, HPC distinguishes itself through its marked biological aggressiveness, propensity for local recurrence, and potential to metastasize beyond the cranial vault [7,8]. HPCs represent a rare entity, comprising merely 0.4% of all primary CNS tumors, in stark contrast to meningiomas, which are 40-50 times more common [9-11].

HPCs are almost invariably associated with the dura mater, demonstrating a pronounced propensity for both local recurrence and metastasis beyond the central nervous system, with recurrence often occurring prior to metastasis [3]. Unlike meningiomas, extracranial manifestations of HPCs are more common. The highly vascular nature of HPCs poses considerable treatment challenges, particularly in pediatric patients due to their lower total blood volume. Surgical intervention requires meticulous management of the tumor's blood supply, with preoperative endovascular embolization often advised. In infants, HPC typically follows a more benign trajectory, showing responsiveness to chemotherapy and even a propensity for spontaneous regression. However, the clinical behavior of HPC in children older than one year aligns more closely with that observed in adult cases [12,13].

## Materials and methods

This study presents an analysis of 14 ISFTs that were surgically treated between 2014 and 2022 at the Department of Neurosurgery, Institutul National de Neurologie si Boli Neurovasculare (National Institute of Neurology and Neurovascular Diseases) in Bucharest, Romania. The study was approved by the Ethics Committee of the National Institute of Neurology and Neurovascular Diseases (approval number: 2/2024) and adhered to the principles outlined in the Declaration of Helsinki. Data handling was conducted in compliance with current General Data Protection Regulation (GDPR) regulations and informed consent was obtained from all patients involved.

Each case included in this study was histologically confirmed as SFT and exhibited at least one pathological feature associated with aggressive behavior: hypercellularity, presence of ≥4 mitoses per 10 high-power fields (HPFs), cytological atypia/pleomorphism, tumor necrosis, infiltrative margins, and/or tumor size ≥10cm. A systematic investigation was conducted, focusing on specific parameters throughout the preoperative, intraoperative, and postoperative phases of patient care, especially surgical characteristics such as tumor localization and dimensions correlated with the type of neurosurgical approach chosen. The possibility of relapse of such tumors was taken into account as well as overall postoperative evolution. Clinical information including gender, tumor site, localization, surgical interventions, and radiotherapy, as well as follow-up details such as recurrence, metastases, and final outcomes, were extracted from relevant files.

Statistical analysis and figure plotting were conducted using Python version 3.10 (Python Software Foundation, Wilmington, Delaware, United States). This work involved the use of Python libraries such as Pandas, NumPy, Seaborn, and Matplotlib.

## Results

**Figure 1:**
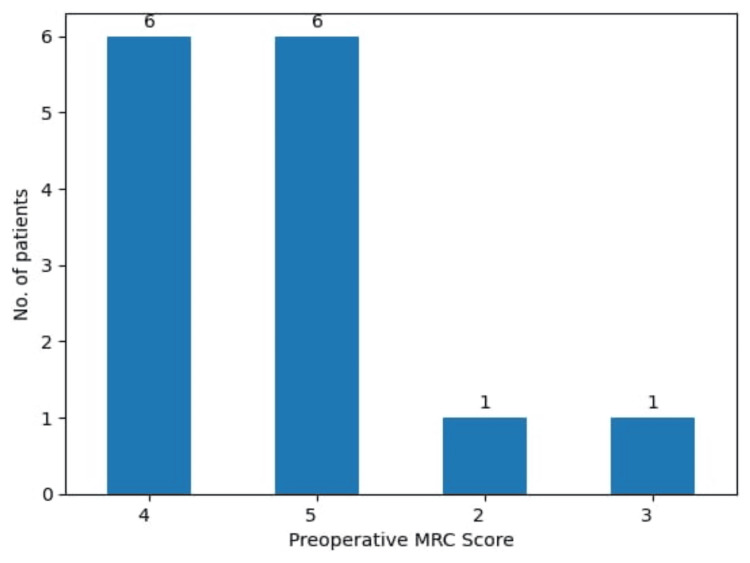
Distribution of study participants according to preoperative MRC score MRC: Medical Research Council Scale for Muscle Strength

The study presents 14 patients with proven histological diagnosis of SFT of which, 64.3% were female and 35.7% were male. Neurological examinations showed that 35.7% had no deficits (Glasgow Coma Scale (GCS) 15), 35.7% had mild deficits (GCS 14), and 28.6% had significant deficits (GCS 13). Most patients had varying degrees of hemiparesis, which were assessed by the MRC Scale (Figure 1); 42.8% had a score of 5, another 42.8% had an MRC score of 4, and two patients had more significant motor deficits, falling under MRC scores of 3 (7.1%) and 2 (7.1%), respectively.

At admission, symptoms among patients included aphasia in one patient (7.15%), temporospatial disorientation in two patients (14.3%), and mild disturbances of consciousness in five patients (35.7%).

**Figure 2:**
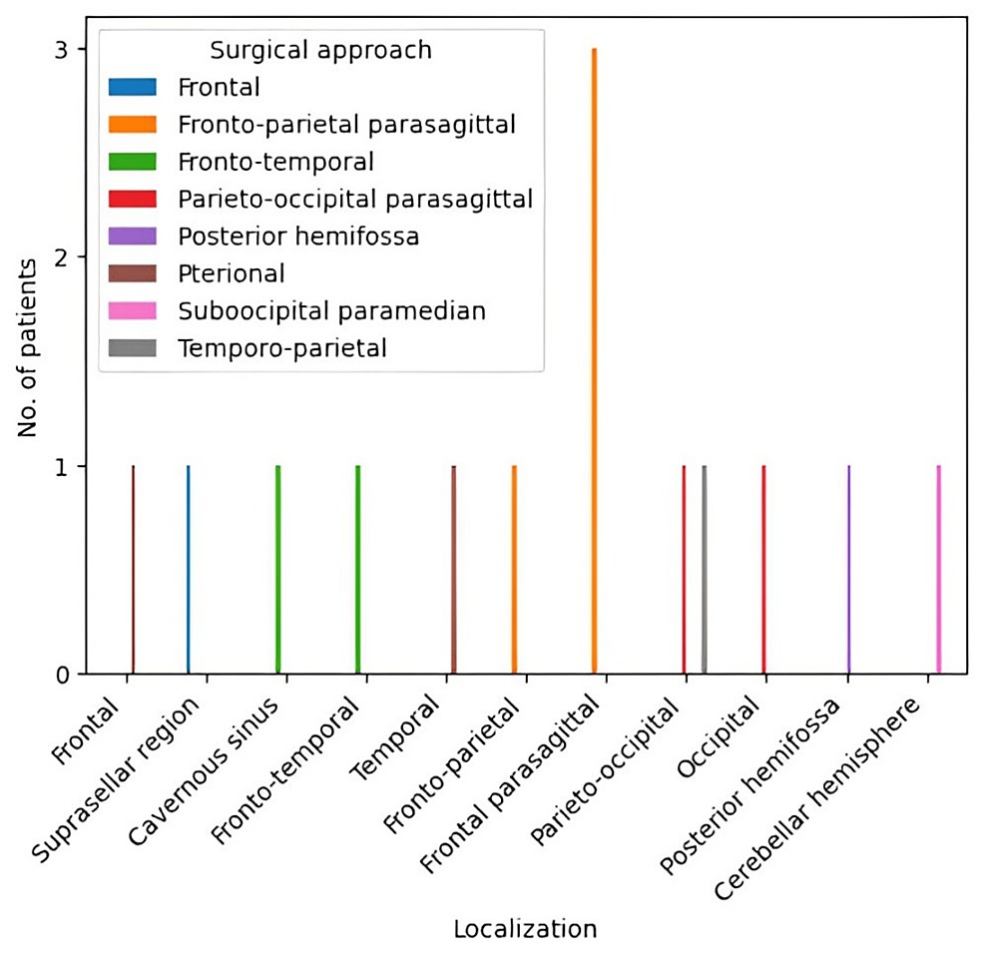
Surgical approach according to localization of the SFT SFT: solitary fibrous tumor

**Figure 3:**
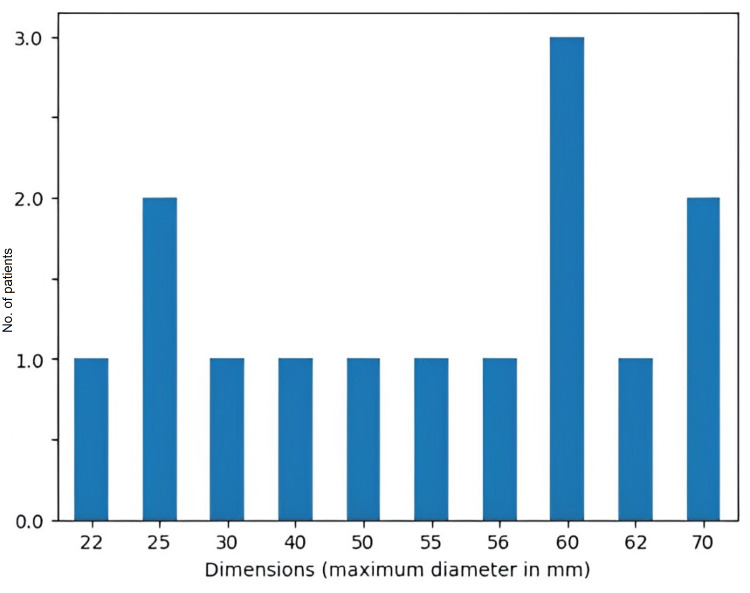
Distribution of study participants according to dimensions (maximum diameter) of the tumors mean: 48.93; std: 17.15; min: 22.00; 25%: 32.50; 50%: 55.50; 75%: 60.00; max: 70.00

The solitary fibrous tumor characteristics investigated in our study encompassed localization and dimensions (maximum diameter) of the tumor. The location of tumors was diverse and difficult to approach surgically, and the median diameter of the tumor was 49 mm (with a range between 22 mm and 70 mm) (Figures 2, 3). Patients underwent surgical treatment. In most of the cases (64.2%,) gross total resection (GTR) was performed. Depending on the location of the tumor, the therapeutic and surgical approaches were different for each patient. Due to the diverse location of the tumors in this study, the surgeon opted to use different surgical approaches, optimal for each case.

**Figure 4:**
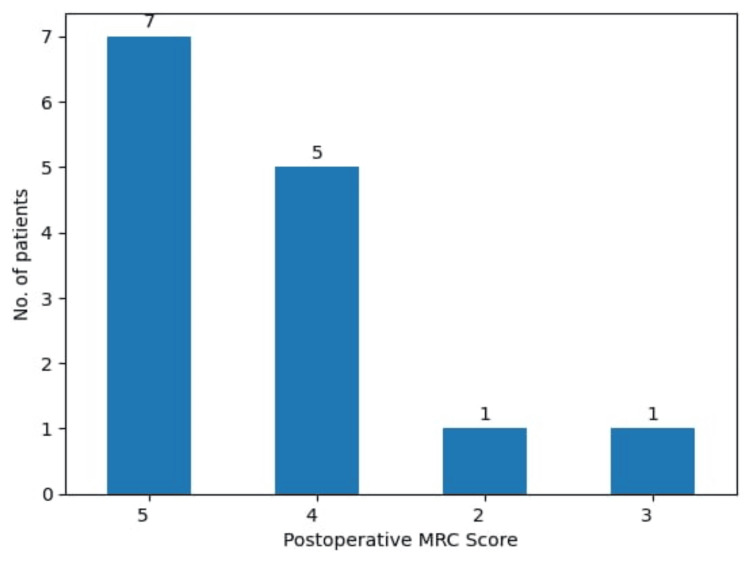
Postoperative MRC scores in the study participants.

After surgery and postoperative recovery, patients had an impressive improvement in neurological status, with only one patient experiencing postoperative complications (significant visual deficit). Unfortunately, most patients retained their motor deficits after surgery, receiving a postoperative MRC score (Figure 4).

Each patient was recommended radiotherapy. Of these, only two patients (14.3%) chose to continue their recovery with radiotherapy.

**Figure 5:**
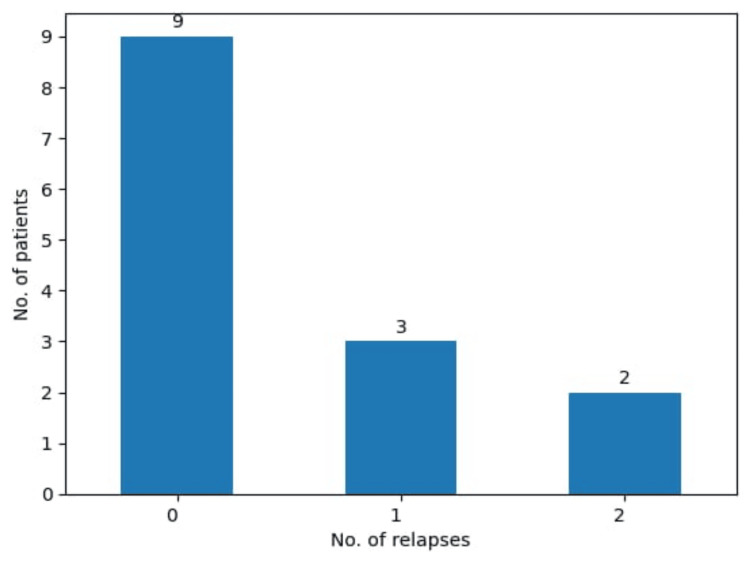
Number of relapses presented in this study.

Despite the special therapeutic care, surgical technique and postoperative recovery, solitary fibrous tumors in literature show a certain degree of relapse. So, cases of relapse are also present in this study (Figure 5). In three patients (21.4%) the tumor recurred only once, and in two patients (14.3%), the tumor recurred two times. Even in one of the two patients who chose radiotherapy, the tumor recurred, underscoring the aggressive nature of the tumor.

In this study, GTR was accomplished in 11 patients, whereas subtotal resection (STR) was executed in three patients, primarily due to the tumor's proximity to neurologically critical regions. Consequently, complete surgical resection was achieved in nine out of the 14 cases, resulting in a success rate of 64.28%.

Illustrative case

A 73-year-old male was hospitalized in our clinic for balance disorders and intracranial hypertension syndrome, with a slowly progressive evolution for about one year. The neurological examination revealed a neocerebellar syndrome.

Initial diagnostic procedures included a brain CT scan which revealed the presence of a tumor that appeared isodense with the surrounding brain tissue. This tumor was located at the level of the left posterior fossa and the pontocerebellar angle on the left side. The mass exerted a marked effect on the IV ventricle, leading to moderate obstructive internal hydrocephalus characterized by minimal periventricular resorption, indicating fluid accumulation due to the obstruction.

**Figure 6:**
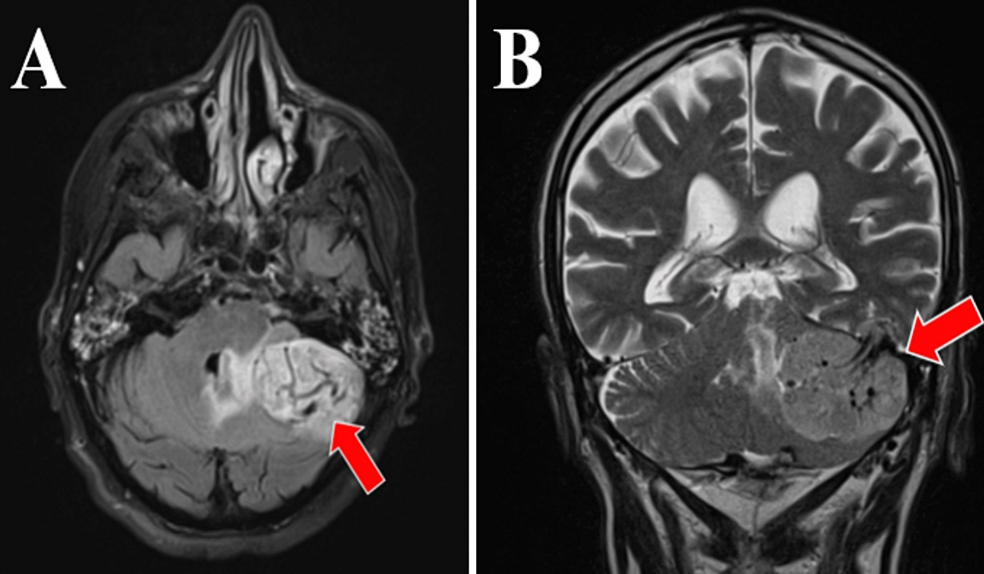
Preoperative MRI: T2, TIRM dark fluid sequence, axial section (A) and T2 native sequence, coronal section (B). MRI T2 TIRM dark fluid sequence, axial section, depict a tumor hyperintense in this sequence at the level of the left posterior fossa and the pontocerebellar angle on the left side. TIRM: turbo inversion recovery magnitude

**Figure 7:**
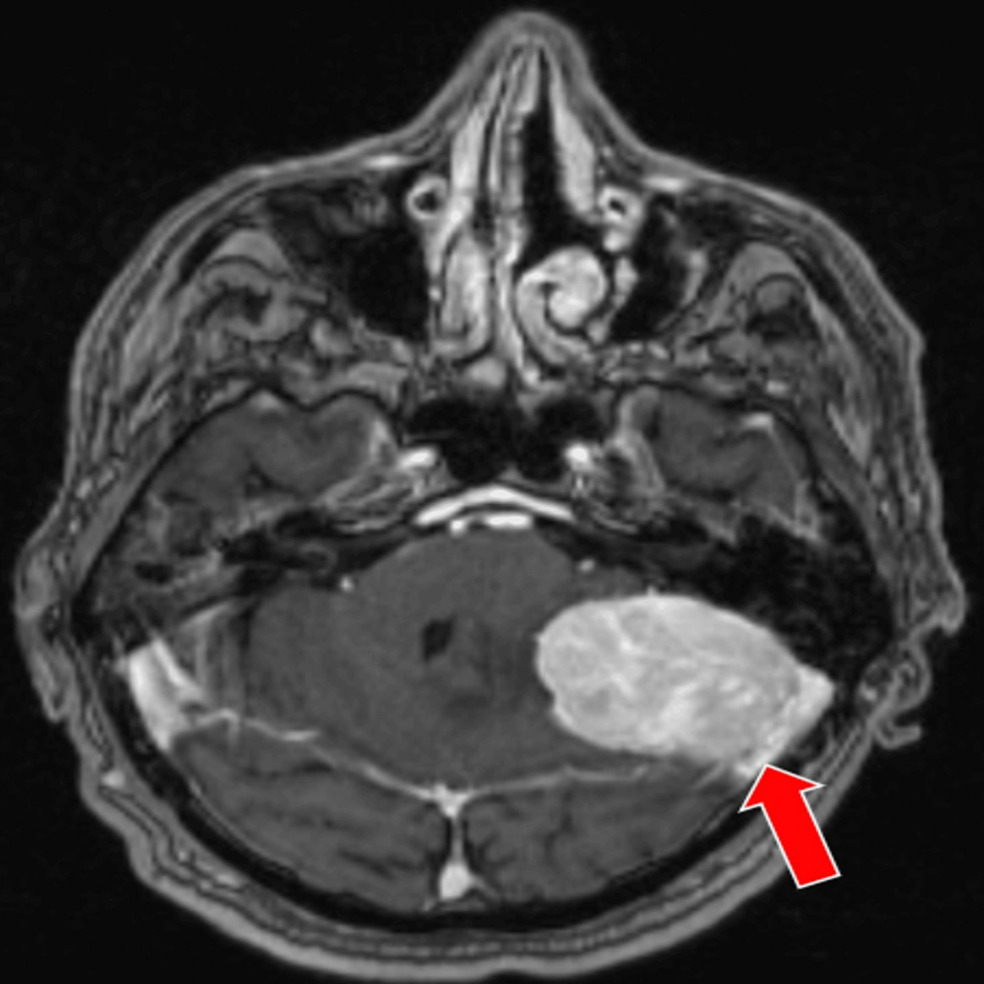
Preoperative MRI T1 MPRAGE sequence, axial section shows that tumor is hyperintense and it can be clearly delineated MPRAGE: Magnetization-Prepared Rapid Acquisition Gradient Echo

To further assess the nature and extent of the tumor, a brain MRI with contrast was performed. This MRI revealed a tumor formation measuring approximately 5 x 6 cm. The tumor was isointense with the brain in T1 and moderately hyperintense in T2 (Figures 6, 7).

**Figure 8:**
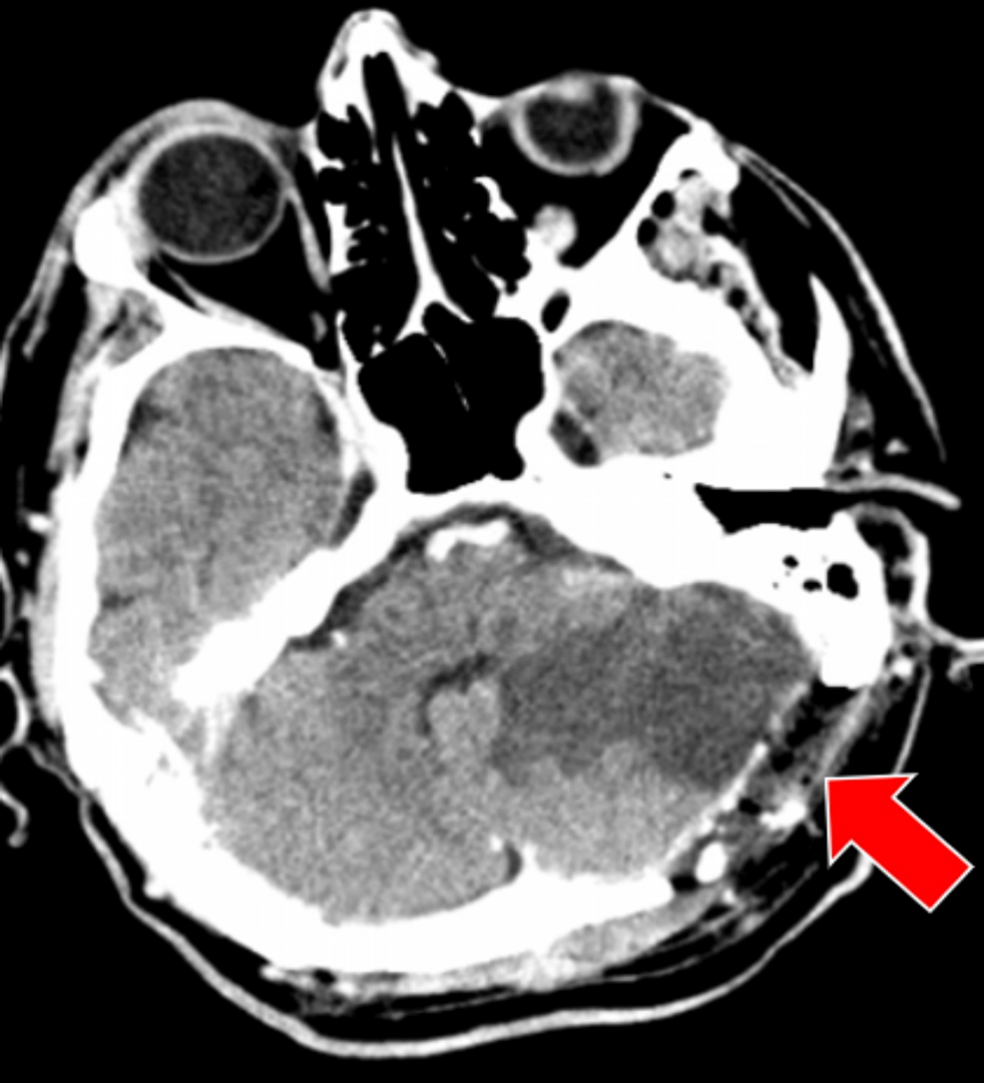
Postoperative CT scan, axial section, that confirmed the gross total resection of the tumor

**Figure 9:**
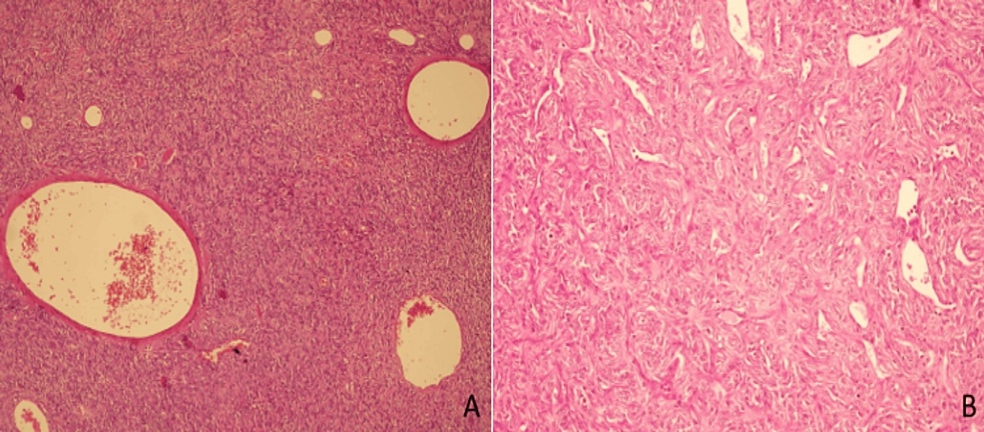
(A) Very high cellularity of the tumor showing a sarcomatous pattern, with large vascular spaces; (B) More dense, smaller vascular profiles taking the so-called “staghorn” shape.

Surgical intervention was performed and ablation of the tumor that had the insertion at the level of the tentorium on the left side was achieved. A postoperative CT scan was done to be sure that the surgical intervention went as expected (Figure 8). A histopathological examination of the tumor was performed and the result was ISFT (Figure 9).

Postoperatively, the evolution was favorable with significant neurological improvement. 

## Discussion

In the 2016 WHO classification for CNS tumors, the entity known as SFT/HPC was classified under a unified designation, yet differentiated by a three-tier grading system [14]. By the 2021 WHO classification update, the approach of applying grading within specific tumor types had been expanded across multiple categories [15]. This adjustment was implemented for various reasons: firstly, to enhance the flexibility of applying grades in relation to the specific tumor type; secondly, to highlight the biological similarities among tumors within the same category, as opposed to focusing solely on their estimated clinical behaviors; and thirdly, to align the grading system for CNS tumors with the grading conventions used for WHO classifications of tumors in other parts of the body [16].

HPC has been discontinued in favor of the singular term of SFT, moving away from the combined nomenclature of SFT/HPC utilized in the 2016 CNS classification [14,15]. This terminology now corresponds with that used for soft tissue tumors, maintaining consistency across classifications. Despite this terminological alignment, a distinct three-tiered grading system specific to the CNS context has been retained, marking a site-associated differentiation in the classification approach [17].

Despite the relatively low occurrence rate of ISFT, precise diagnosis is crucial to enhance the effectiveness of clinical management and treatment planning. It has been observed that high-grade SFTs are prone to local recurrence and/or metastasis [18]. Clinically and radiologically, SFTs present with features that closely resemble those of meningiomas, making differentiation challenging. A distinguishing feature of SFTs is their tendency to manifest at a younger age, typically peaking between the fourth and fifth decades of life, in contrast to the generally later onset of meningioma [19].

The literature contains only a limited number of SFT case reports, with even fewer instances documented as part of surgical series that encompass an adequate volume of cases accompanied by comprehensive follow-up data. Our statistical analysis of the series offers valuable insights; some of these findings are consistent with those previously reported in the literature, while others present divergences from established data.

In terms of gender predilection, several studies have identified a male predominance, as evidenced by the male-to-female ratio [20,21]. Certain studies have noted a female predominance when examining the male-to-female ratio [9,13]. Our series shows a male-to-female ratio of 5:9.

In the present study, GTR was achieved in 11 patients while STR was performed in three patients, attributed to the tumor's proximity to areas critical for neurological function. Consequently, complete surgical resection was accomplished in nine out of 14 cases, translating to a success rate of 64.28%. This outcome is notably favorable when compared to prior surgical cohorts, which have documented total excision rates ranging from 25% to 70% [22]. The superior results observed in our study are likely attributable to the employment of advanced intraoperative technologies aimed at reducing surgical risks. Utilization of the operating microscope and neuronavigation systems has been instrumental in achieving optimal resections while concurrently mitigating the likelihood of postoperative complications, such as deficits or mortality. This aspect is particularly critical considering the established correlation between the extent of resection and prognosis in HPC. Furthermore, the surgical intervention aims not only at tumor removal but also at enhancing the patient's quality of life and prolonging the period of recurrence-free survival, goals that were attainable for the majority of participants in our series.

While studying the literature on SFT/HPC, we delved into studies that presented significant contributions to the field, and some of them have been listed in Table 1.

**Table 1:** Studies that presented significant contributions to the field. N/A: not available; GTR: gross total resection; STR: subtotal resection; PR: partial resection

Study (author, year)	Age (years), median	Sex, number		WHO grade of tumor			Treatment strategy, number			Relapses, number
		M	F	I	II	III	GTR	STR	PR	
Rutkowski et al., 2011 [9]	48	15	20	N/A			16	19	0	18
Melone et al., 2014 [13]	46.9	17	26	0	33	10	30	13	0	18
Yip et al., 2020 [23]	52.8	3	4	0	6	1	1	0	6	7
Chenhui et al., 2021 [24]	55.1	11	6	8	8	1	N/A			5
Bassiouni et al., 2007 [25]	38	7	5	N/A			11	1	0	5

The primary limitations of this current analysis are its retrospective nature, the small patient cohort size, and a relatively brief follow-up period. Additionally, a constraint lies in the absence of SFT lacking unfavorable histological characteristics. Consequently, the study cannot determine the proportion of SFTs devoid of any aggressive histological or molecular features (such as TP53 and/or TERT promoter mutations) that exhibit clinically malignant behavior. 

## Conclusions

SFTs are rare tumors with a versatile history, given the change in their classification over time, and a high recurrence percentage. We have tried to highlight the relevance of GTR in the context of the aggressiveness of this tumor. Thus, the surgeon must take a serious look at the level of resection that can be performed in accordance with the patient's quality of life postoperatively. Moreover, our case series sheds light on the fact that with modern surgical techniques, GTR can be achieved to a higher extent, which in our case was 64.2%.

## References

[1] Cheng L, Ni H, Dai Y (2020). Intracranial solitary fibrous tumor mimicking meningioma: a case report. Medicine (Baltimore).

[2] Carneiro SS, Scheithauer BW, Nascimento AG, Hirose T, Davis DH (1996). Solitary fibrous tumor of the meninges: a lesion distinct from fibrous meningioma. A clinicopathologic and immunohistochemical study. Am J Clin Pathol.

[3] Louis DN, Ohgaki H, Wiestler OD (2007). The 2007 WHO classification of tumours of the central nervous system. Acta Neuropathol.

[4] Ma L, Wang L, Fang X, Zhao CH, Sun L (2018). Diagnosis and treatment of solitary fibrous tumor/hemangiopericytoma of central nervous system. Retrospective report of 17 patients and literature review. Neuro Endocrinol Lett.

[5] Huang Z, Dai D, Tang G (2022). Rare magnetic resonance imaging findings of intracranial solitary fibrous tumor: a case report. Medicine (Baltimore).

[6] Stout AP, Murray MR (1942). Hemangiopericytoma: a vascular tumor featuring Zimmermann's pericytes. Ann Surg.

[7] Choi BH, Moon JI, Baek HJ (2016). Intraventricular anaplastic hemangiopericytoma: CT and MR imaging findings. J Korean Soc Radiol.

[8] Ding D, Sheehan JP (2014). Intracranial hemangiopericytomas: a wolf in sheep's clothing. World Neurosurg.

[9] Rutkowski MJ, Jian BJ, Bloch O (2012). Intracranial hemangiopericytoma: clinical experience and treatment considerations in a modern series of 40 adult patients. Cancer.

[10] Marcó Del Pont F, Ries Centeno T, Villalonga JF, Giovannini SJ, Caffaratti G, Lorefice E, Cervio A (2021). Results in the treatment of intracranial hemangiopericytomas. Case series. Neurocirugia (Astur : Engl Ed).

[11] Chan RC, Thompson GB (1984). Morbidity, mortality, and quality of life following surgery for intracranial meningiomas. A retrospective study in 257 cases. J Neurosurg.

[12] Laviv Y, Michowitz S, Schwartz M (2012). Neonatal intracranial hemangiopericytoma: a 7-year follow-up. Acta Neurochir (Wien).

[13] Melone AG, D'Elia A, Santoro F, Salvati M, Delfini R, Cantore G, Santoro A (2014). Intracranial hemangiopericytoma--our experience in 30 years: a series of 43 cases and review of the literature. World Neurosurg.

[14] Louis DN, Perry A, Reifenberger G (2016). The 2016 World Health Organization Classification of Tumors of the Central Nervous System: a summary. Acta Neuropathol.

[15] Louis DN, Perry A, Wesseling P (2021). The 2021 WHO Classification of Tumors of the Central Nervous System: a summary. Neuro Oncol.

[16] Louis DN, von Deimling A (2017). Grading of diffuse astrocytic gliomas: Broders, Kernohan, Zülch, the WHO… and Shakespeare. Acta Neuropathol.

[17] Torp SH, Solheim O, Skjulsvik AJ (2022). The WHO 2021 Classification of Central Nervous System tumours: a practical update on what neurosurgeons need to know-a minireview. Acta Neurochir (Wien).

[18] Sibtain NA, Butt S, Connor SE (2007). Imaging features of central nervous system haemangiopericytomas. Eur Radiol.

[19] Yamashita D, Suehiro S, Kohno S (2021). Intracranial anaplastic solitary fibrous tumor/hemangiopericytoma: immunohistochemical markers for definitive diagnosis. Neurosurg Rev.

[20] Guthrie BL, Ebersold MJ, Scheithauer BW, Shaw EG (1989). Meningeal hemangiopericytoma: histopathological features, treatment, and long-term follow-up of 44 cases. Neurosurgery.

[21] Mena H, Ribas JL, Pezeshkpour GH (1991). Hemangiopericytoma of the central nervous system: a review of 94 cases. Hum Pathol.

[22] Ecker RD, Marsh WR, Pollock BE, Kurtkaya-Yapicier O, McClelland R, Scheithauer BW, Buckner JC (2003). Hemangiopericytoma in the central nervous system: treatment, pathological features, and long-term follow up in 38 patients. J Neurosurg.

[23] Yip CM, Hsu SS, Liao WC (2020). Intracranial solitary fibrous tumor/hemangiopericytoma - a case series. Surg Neurol Int.

[24] Chenhui Z, He G, Wu Z (2021). Intracranial solitary fibrous tumor/hemangiopericytomas: a clinical analysis of a series of 17 patients. Br J Neurosurg.

[25] Bassiouni H, Asgari S, Hübschen U, König HJ, Stolke D (2007). Intracranial hemangiopericytoma: treatment outcomes in a consecutive series. Zentralbl Neurochir.

